# Supplementary material for: Genomic evidence for the Old divergence of Southern European wolf populations
Source: Proc Biol Sci. 2020 Jul 22;287(1931):20201206. doi: 10.1098/rspb.2020.1206 (PMC7423677; doi:10.1098/rspb.2020.1206)
Supplement: Supplementary command lines and tables [file rspb20201206supp1.pdf]

ELECTRONIC SUPPLEMENTARY MATERIAL

Genomic Evidence for the Old Divergence of Southern European Wolf Populations

Pedro Silva, Marco Galaverni, Diego Ortega-Del Vecchyo, Zhenxin Fan, Romolo Caniglia, Elena Fabbri, Ettore Randi, Robert Wayne, Raquel Godinho

COMMAND LINES FOR SIMULATED DATA

- **Command Line 1**: ms (Hudson, 2002) simulation example using the inferred demographic parameters by G-PhoCS in the main analysis. Simulated trees are written to the plain text file 'treefile.txt'.

```
./ms 19 15000 -r 919.08 1000 -I 10 1 2 2 2 2 2 2 2 2 2 -n 1 0.0003114 -n 2
0.0001538 -n 3 0.0000642 -n 4 0.0003658 -n 5 0.0019099 -n 6 0.0004691 -n 7
0.0001650 -n 8 0.0004773 -n 9 0.0001588 -n 10 0.0014978 -m 8 1 14492.72 -m 8
2 2898.55 -m 8 3 1449.28 -m 5 2 3896.10 -m 3 5 2331.61 -m 7 9 2173.91 -m 4 5
134.18 -m 4 3 1554.40 -ej 0.0000138 8 9 -em 0.0000138 8 1 0.0 -em 0.0000138 8
2 0.0 -em 0.0000138 8 3 0.0 -em 0.0000138 7 9 0.0 -en 0.0000138 9 0.0001162 -
ej 0.0000228 7 9 -en 0.0000228 9 0.0004047 -ej 0.0000239 6 9 -en 0.0000239 9
0.0015784 -ej 0.0000385 2 1 -em 0.0000385 5 2 0.0 -en 0.0000385 1 0.0000022 -
ej 0.0000386 3 1 -em 0.0000386 3 5 0.0 -em 0.0000386 4 3 0.0 -en 0.0000386 1
0.0004778 -ej 0.0000745 5 9 -em 0.0000745 4 5 0.0 -en 0.0000745 9 0.0001719 -
ej 0.0000749 4 9 -en 0.0000749 9 0.0003849 -ej 0.000080 9 1 -em 0.000080 10 1
555.99 -en 0.000080 1 0.0037788 -ej 0.003497 10 1 -em 0.003497 10 1 0.0 -en
0.003497 1 0.00047172 -T -seeds 1 2 3 -p 10 | tail -n +4 | grep -v '//' >
treefile.txt
```

- **Command Line 2**: Example of how to simulate sequence data from a set of trees (in 'treefile.txt') using seq-gen (Rambaut & Grassly, 1997). The resulting sequences are written to the plain text file 'loci.txt'.

```
./seq-gen -mHKY -l 1000 -s 0.4 -fe -t 0.5 -p 1000 < treefile.txt > loci.txt
```

- **Command Line 3**: ms simulation example using a demographic history where the Iberian wolves (Portuguese and Spanish wolves) diverged more recently (90% of the time inferred in the main analysis). This command is similar to Command Line 1 above but the first merging event (backwards in time) occurs at time 0.00000138 instead of 0.0000138

```
./ms 19 15000 -r 919.08 1000 -I 10 1 2 2 2 2 2 2 2 2 2 -n 1 0.0003114 -n 2
0.0001538 -n 3 0.0000642 -n 4 0.0003658 -n 5 0.0019099 -n 6 0.0004691 -n 7
0.0001650 -n 8 0.0004773 -n 9 0.0001588 -n 10 0.0014978 -m 8 1 14492.72 -m 8
2 2898.55 -m 8 3 1449.28 -m 5 2 3896.10 -m 3 5 2331.61 -m 7 9 2173.91 -m 4 5
134.18 -m 4 3 1554.40 -ej 0.00000138 8 9 -em 0.00000138 8 1 0.0 -em
0.00000138 8 2 0.0 -em 0.00000138 8 3 0.0 -em 0.00000138 7 9 0.0 -en
0.00000138 9 0.0001162 -ej 0.0000228 7 9 -en 0.0000228 9 0.0004047 -ej
0.0000239 6 9 -en 0.0000239 9 0.0015784 -ej 0.0000385 2 1 -em 0.0000385 5 2
0.0 -en 0.0000385 1 0.0000022 -ej 0.0000386 3 1 -em 0.0000386 3 5 0.0 -em
0.0000386 4 3 0.0 -en 0.0000386 1 0.0004778 -ej 0.0000745 5 9 -em 0.0000745 4
5 0.0 -en 0.0000745 9 0.0001719 -ej 0.0000749 4 9 -en 0.0000749 9 0.0003849 -
ej 0.000080 9 1 -em 0.000080 10 1 555.99 -en 0.000080 1 0.0037788 -ej
0.003497 10 1 -em 0.003497 10 1 0.0 -en 0.003497 1 0.00047172 -T -seeds 1 2 3
-p 10 | tail -n +4 | grep -v '//' > treefile2.txt
```

- **Command Line 4**: ms simulation example using a demographic history where the Iberian wolves (Portuguese and Spanish wolves) belong to the same, panmictic population. This command is similar to Command Line 1 above but the first merging event (backwards in time) occurs at time 0.

```
./ms 19 15000 -r 919.08 1000 -I 10 1 2 2 2 2 2 2 2 2 2 -n 1 0.0003114 -n 2
0.0001538 -n 3 0.0000642 -n 4 0.0003658 -n 5 0.0019099 -n 6 0.0004691 -n 7
0.0001650 -n 8 0.0004773 -n 9 0.0001588 -n 10 0.0014978 -m 8 1 14492.72 -m 8
2 2898.55 -m 8 3 1449.28 -m 5 2 3896.10 -m 3 5 2331.61 -m 7 9 2173.91 -m 4 5
134.18 -m 4 3 1554.40 -ej 0.0 8 9 -em 0.0 8 1 0.0 -em 0.0 8 2 0.0 -em 0.0 8 3
0.0 -em 0.0 7 9 0.0 -en 0.0 9 0.0001162 -ej 0.0000228 7 9 -en 0.0000228 9
0.0004047 -ej 0.0000239 6 9 -en 0.0000239 9 0.0015784 -ej 0.0000385 2 1 -em
0.0000385 5 2 0.0 -en 0.0000385 1 0.0000022 -ej 0.0000386 3 1 -em 0.0000386 3
```

```
5 0.0 -em 0.0000386 4 3 0.0 -en 0.0000386 1 0.0004778 -ej 0.0000745 5 9 -em
0.0000745 4 5 0.0 -en 0.0000745 9 0.0001719 -ej 0.0000749 4 9 -en 0.0000749 9
0.0003849 -ej 0.000080 9 1 -em 0.000080 10 1 555.99 -en 0.000080 1 0.0037788
-ej 0.003497 10 1 -em 0.003497 10 1 0.0 -en 0.003497 1 0.00047172 -T -seeds 1
2 3 -p 10 | tail -n +4 | grep -v '//' > treefile3.txt
```

SUPPLEMENTARY TABLES

Supplementary Table S1: Parameter priors for G-PhoCS analyses. Only values differing from defaults are listed. ancDOG1: ancestor of boxer and basenji; ancDOG: ancestor of dogs (boxer, basenji, dingo); ancIB: ancestor of Iberian wolves (Portuguese and Spanish); ancIT-IB: ancestor of Italian and Iberian wolves; ancCR-IT-IB: ancestor of Croatian, Italian and Iberian wolves; ancIS-CR-IT-IB: ancestor of Israeli, Croatian, Italian and Iberian wolves; ancWOLF: ancestor of wolves; ancDW: ancestor of dogs and wolves; root: ancestor of all sampled canids in this study.

parameter	value	applied to	description
locus-mut-rate	VAR 1.0	all	defines variable mutation rates across loci; 1.0 is the α parameter for the Dirichlet prior for the rate variation
find-finetunes	TRUE	all	invokes automatic finetuning procedure for several MCMC parameters
tau-theta-alpha	1.0	all	α parameter for Gamma distribution used for priors of all τ and θ parameters
tau-theta-beta	10000	all	β parameter for Gamma distribution used for priors of all τ and θ parameters
tau-theta-print	10000	all	scales up final τ and θ parameters by 10^4 for better readability
mig-rate-alpha	0.002	all	α parameter for Gamma distribution used for priors of all migration rate
mig-rate-beta	0.00001	all	β parameter for Gamma distribution used for priors of all migration rates
mig-rate-print	0.001	all	scales down final migration parameter by 10^{-3} for better readability
tau-initial	0.00002	ancDOG1	initial τ around which to start the MCMC; the actual value is sampled uniformly in the interval [0.8, 1.2] x tau-initial
tau-initial	0.00003	ancDOG	
tau-initial	0.00002	ancIB	
tau-initial	0.00002	ancIT-IB	
tau-initial	0.00003	ancCR-IT-IB	
tau-initial	0.00004	ancIS-CR-IT-IB	
tau-initial	0.00004	ancWOLF	
tau-initial	0.00005	ancDW	
tau-initial	0.00005	root	

Supplementary Table S2: **Demographic parameter estimates by G-PhoCS**. Raw estimates are scaled up by 10^4 for better readability. Calibrated estimates (in number of individuals and years) are provided assuming two combinations of mutation rates and generation times (see Methods). N: effective population size; T: time; BOX: boxer; BAS: basenji; DIN: dingo; ISW: Israeli wolf; CRW: Croatian wolf; ITW: Italian wolf; SPW: Spanish wolf; PTW: Portuguese wolf; JAC: golden jackal; ancDOG1: ancestor of boxer and basenji; ancDOG: ancestor of dogs (boxer, basenji, dingo); ancIB: ancestor of Iberian wolves (Portuguese and Spanish); ancIT-IB: ancestor of Italian and Iberian wolves; ancCR-IT-IB: ancestor of Croatian, Italian and Iberian wolves; ancIS-CR-IT-IB: ancestor of Israeli, Croatian, Italian and Iberian wolves; ancWOLF: ancestor of wolves; ancDW: ancestor of dogs and wolves; root: ancestor of all sampled canids in this study.

**parameter**	**raw estimate x10^4**	**mutation rate: $4x10^{-9}$ /bp/generation generation time: 4.5 years**	**mutation rate: $1x10^{-8}$/bp/generation generation time: 3 years**
N_{BOX}	1.25 (0.03-3.07)	7786 (216-19171)	3115 (86-7669)
N_{BAS}	0.62 (0.53-0.7)	3846 (3323-4369)	1538 (1329-1748)
N_{DIN}	0.26 (0.21-0.33)	1606 (1309-2033)	643 (524-813)
N_{CHW}	1.46 (1.29-1.63)	9146 (8076-10194)	3658 (3230-4078)
N_{ISW}	7.64 (6.14-9.24)	47748 (38361-57740)	19099 (15344-23096)
N_{CRW}	1.88 (1.33-2.36)	11728 (8286-14743)	4691 (3314-5897)
N_{ITW}	0.66 (0.46-0.82)	4124 (2887-5104)	1650 (1155-2042)
N_{SPW}	1.91 (0.67-3.15)	11933 (4178-19689)	4773 (1671-7876)
N_{PTW}	0.64 (0.3-0.9)	3969 (1890-5656)	1588 (756-2262)
N_{JAC}	5.99 (5.73-6.25)	37444 (35827-39048)	14978 (14331-15619)
$N_{ancDOG1}$	0.01 (0-0.02)	56 (15-129)	22 (6-52)
N_{ancDOG}	1.91 (1.64-2.21)	11944 (10234-13813)	4778 (4094-5525)
N_{ancIB}	0.46 (0.26-0.81)	2906 (1637-5040)	1162 (655-2016)
$N_{ancIT-IB}$	1.62 (0.14-4.28)	10117 (848-26774)	4047 (339-10710)
$N_{ancCR-IT-IB}$	6.31 (5.05-7.78)	39460 (31554-48599)	15784 (12622-19440)
$N_{IS-CR-IT-IB}$	0.69 (0.05-2.2)	4298 (333-13727)	1719 (133-5491)
$N_{ancWOLF}$	1.54 (0.52-2.79)	9622 (3228-17458)	3849 (1291-6983)
N_{ancDW}	15.12 (14.81-15.43)	94471 (92586-96436)	37789 (37034-38574)
N_{root}	1.89 (0-6.07)	11793 (2-37934)	4717 (1-15174)
$T_{ancDOG1}$	0.15 (0.14-0.17)	17325 (15311-19451)	4620 (4083-5187)
T_{ancDOG}	0.15 (0.14-0.17)	17370 (15345-19530)	4632 (4092-5208)
T_{ancIB}	0.06 (0.03-0.08)	6210 (3071-8753)	1656 (819-2334)
$T_{ancIT-IB}$	0.09 (0.06-0.11)	10271 (7211-12229)	2739 (1923-3261)
$T_{ancCR-IT-IB}$	0.1 (0.07-0.11)	10766 (7920-12668)	2871 (2112-3378)
$T_{IS-CR-IT-IB}$	0.3 (0.27-0.32)	33536 (30713-36304)	8943 (8190-9681)
$T_{ancWOLF}$	0.3 (0.28-0.32)	33705 (31016-36338)	8988 (8271-9690)
T_{ancDW}	0.32 (0.3-0.35)	36000 (33480-38858)	9600 (8928-10362)
T_{root}	13.99 (11.02-15.58)	1573808 (1239615-1752615)	419682 (330564-467364)

Supplementary Table S3: Migration estimates by G-PhoCS. Raw estimates are scaled down by 10^{-3} for better readability. Calibrated estimates are calculated based on raw divergence time estimates (Supplementary Table S2) and are independent from assumptions of mutation rate or generation time.

**migration direction**	**raw estimate (M) x10^{-3}**	**calibrated estimate (m_{tot})**
BOX->SPW	29.33 (4.77-73.08)	0.2 (0.03-0.49)
BAS->SPW	6.29 (0-12.13)	0.04 (0-0.08)
DIN->SPW	2.85 (0-5.9)	0.02 (0-0.04)
BAS->ISW	7.03 (5.39-8.59)	0.15 (0.12-0.19)
ISW->DIN	4.15 (0-8.36)	0.09 (0-0.18)
PTW->ITW	4.6 (0-15.58)	0.03 (0-0.1)
ancDW->JAC	1.39 (1.3-1.46)	1.9 (1.78-2)
CHW->ancWOLF	19.28 (0-127.61)	0 (0-0.02)
ISW->CHW	0.28 (0-1.98)	0.01 (0-0.05)
DIN->CHW	2.88 (1.87-3.99)	0.06 (0.04-0.09)

Supplementary Table S4: Comparison of demographic parameter estimates by G-PhoCS from simulated data. Only raw estimates are shown, scaled up by 10^4 in the case of effective population sizes (N) and times (T), and scaled down by 10^{-3} in the case of migration rates (M). Parameters are the same as in Supplementary Tables S2 and S3.

**parameter**	**control simulation**	**smaller divergence time PTW-SPW**	**PTW-SPW panmixia**
N_{BOX}	1.93 (0.88-3.08)	2.01 (0.03-4.41)	1.04 (0-3)
N_{BAS}	0.86 (0.79-0.93)	0.94 (0.79-1.08)	0.45 (0.37-0.53)
N_{DIN}	0.38 (0.3-0.44)	0.37 (0.26-0.48)	0.34 (0.26-0.41)
N_{CHW}	1.45 (1.31-1.57)	0.89 (0.68-1.2)	1.5 (1.36-1.63)
N_{ISW}	7.32 (6.09-8.61)	5.49 (3.85-7.61)	7.4 (6.23-8.63)
N_{CRW}	2.77 (2.24-3.26)	2.06 (1.31-2.66)	1.91 (1.27-2.42)
N_{ITW}	1.09 (0.91-1.27)	0.67 (0.48-0.86)	0.67 (0.44-0.83)
N_{SPW}	3.9 (2.81-5.16)	1.39 (0.18-3.2)	1.55 (0-3.75)
N_{PTW}	1.26 (1.09-1.43)	1.83 (0.22-4)	1.22 (0.01-3.25)
N_{JAC}	7.2 (6.95-7.44)	7.35 (7.11-7.61)	7.4 (7.15-7.65)
$N_{ancDOG1}$	0.01 (0.01-0.02)	0.11 (0.01-0.37)	1.17 (0.33-2.04)
N_{ancDOG}	0.82 (0.7-0.94)	1.27 (0.68-2.01)	2.18 (1.54-2.8)
N_{ancIB}	0.45 (0.2-0.74)	0.48 (0.3-0.62)	0.47 (0.32-0.58)
$N_{ancIT-IB}$	1.89 (0.15-4.48)	1.97 (0.08-4.59)	2.17 (0.04-5.1)
$N_{ancCR-IT-IB}$	3.96 (3.05-4.76)	2.23 (0.72-5.29)	6.93 (5.84-8.49)
$N_{IS-CR-IT-IB}$	1.07 (0-3.63)	2.4 (0.22-5.69)	1.68 (0.03-4.94)
$N_{ancWOLF}$	0.29 (0.06-0.59)	16.16 (7.93-23.95)	1.41 (0.13-3.66)
N_{ancDW}	18.17 (17.86-18.5)	18.12 (17.79-18.46)	18.48 (18.19-18.78)
N_{root}	0.64 (0-1.83)	0.18 (0-1.5)	1.08 (0.16-2.24)
$T_{ancDOG1}$	0.21 (0.2-0.23)	0.24 (0.2-0.28)	0.11 (0.09-0.13)
T_{ancDOG}	0.21 (0.2-0.23)	0.24 (0.21-0.28)	0.17 (0.14-0.21)
T_{ancIB}	0.11 (0.11-0.12)	0.01 (0-0.02)	0 (0-0.01)
$T_{ancIT-IB}$	0.15 (0.12-0.17)	0.09 (0.07-0.12)	0.09 (0.06-0.11)
$T_{ancCR-IT-IB}$	0.15 (0.12-0.17)	0.1 (0.06-0.12)	0.1 (0.06-0.12)
$T_{IS-CR-IT-IB}$	0.28 (0.26-0.29)	0.17 (0.13-0.23)	0.31 (0.29-0.33)
$T_{ancWOLF}$	0.28 (0.26-0.29)	0.17 (0.13-0.23)	0.31 (0.29-0.34)
T_{ancDW}	0.28 (0.26-0.3)	0.35 (0.31-0.39)	0.33 (0.31-0.35)
T_{root}	12.43 (11.7-12.94)	12.64 (11.86-12.94)	12.05 (11.31-12.62)
mBOX->SPW	0.22 (0.17-0.28)	0.04 (0-0.09)	0 (0-0)
mBAS->SPW	0.02 (0-0.05)	0 (0-0.02)	0 (0-0)
mDIN->SPW	0.02 (0-0.03)	0 (0-0)	0 (0-0)
mBAS->ISW	0.13 (0.1-0.15)	0.13 (0.09-0.17)	0.15 (0.13-0.16)
mISW->DIN	0.05 (0-0.17)	0.11 (0-0.23)	0 (0-0.01)
mPTW->ITW	0.02 (0-0.06)	0 (0-0.02)	0 (0-0)
mancDW->JAC	1.19 (1.14-1.24)	1.22 (1.17-1.27)	1.16 (1.13-1.2)
mCHW->ancWOLF	0 (0-0.01)	0.01 (0-0.04)	0 (0-0.01)
mISW->CHW	0.01 (0-0.04)	0.01 (0-0.05)	0 (0-0.01)
mDIN->CHW	0.04 (0.03-0.06)	0.05 (0.03-0.06)	0.07 (0.05-0.09)

Supplementary Table S5: Comparison of demographic parameter estimates by G-PhoCS to assess the influence of dog ancestry in the Spanish wolf (SPW). Only raw estimates are shown, scaled up by 10^4 in the case of effective population sizes (N) and times (T), and scaled down by 10^{-3} in the case of migration rates (M). Results from the main analysis are recapitulated (same as Supplementary Tables S2 and S3), in addition to analyses without the Spanish wolf, with 'dog-like' genomic regions removed from the Spanish wolf genome, and with random regions in the same proportion removed from the Spanish wolf. Parameters are the same as in Supplementary Tables S2 and S3.

parameter	main analysis	without SPW	'dog-like' regions removed from SPW	random regions removed from SPW
N_{BOX}	1.25 (0.03-3.07)	1.02 (0.01-3.06)	0.81 (0-2.62)	0.91 (0-2.2)
N_{BAS}	0.62 (0.53-0.7)	0.77 (0.63-0.95)	0.73 (0.6-0.86)	0.54 (0.44-0.63)
N_{DIN}	0.26 (0.21-0.33)	0.33 (0.28-0.39)	0.39 (0.32-0.45)	0.37 (0.29-0.47)
N_{CHW}	1.46 (1.29-1.63)	1.26 (1.06-1.53)	1 (0.85-1.14)	1.11 (0.87-1.35)
N_{ISW}	7.64 (6.14-9.24)	6.48 (4.98-8.22)	5.47 (4.08-6.92)	5.49 (4.15-6.94)
N_{CRW}	1.88 (1.33-2.36)	1.95 (0.81-2.82)	1.96 (1.47-2.53)	2.96 (2.45-3.49)
N_{ITW}	0.66 (0.46-0.82)	0.69 (0.27-0.99)	0.68 (0.54-0.85)	1.08 (0.92-1.22)
N_{SPW}	1.91 (0.67-3.15)	-	0.6 (0.45-0.77)	3.79 (2.4-5.48)
N_{PTW}	0.64 (0.3-0.9)	0.57 (0.24-0.8)	0.81 (0.6-1.03)	1.63 (1.32-1.97)
N_{JAC}	5.99 (5.73-6.25)	5.98 (5.74-6.22)	6.05 (5.8-6.29)	6.02 (5.75-6.29)
$N_{ancDOG1}$	0.01 (0-0.02)	0.97 (0.26-1.93)	1.66 (0.57-2.8)	2.96 (1.94-4.23)
N_{ancDOG}	1.91 (1.64-2.21)	0.87 (0.37-1.33)	0.6 (0.16-1.02)	0.58 (0.14-0.9)
N_{ancIB}	0.46 (0.26-0.81)	-	0.27 (0.05-0.67)	0.02 (0.01-0.05)
$N_{ancIT-IB}$	1.62 (0.14-4.28)	1.71 (0.03-4.18)	1.48 (0.07-3.59)	1.54 (0.1-3.76)
$N_{ancCR-IT-IB}$	6.31 (5.05-7.78)	4.42 (2.21-7.34)	3.13 (1.98-4.27)	1.6 (0.6-2.87)
$N_{IS-CR-IT-IB}$	0.69 (0.05-2.2)	1.77 (0.02-4.76)	0.22 (0.01-1.13)	2.5 (0.11-5.76)
$N_{ancWOLF}$	1.54 (0.52-2.79)	4.63 (0.66-8.18)	8.95 (5.76-12.03)	7.44 (2.49-11.69)
N_{ancDW}	15.12 (14.81-15.43)	15.11 (14.74-15.49)	15.24 (14.91-15.59)	15.24 (14.86-15.61)
N_{root}	1.89 (0-6.07)	0.87 (0-4.55)	3.55 (0-6.02)	2.83 (0.01-6.12)
$T_{ancDOG1}$	0.15 (0.14-0.17)	0.19 (0.16-0.24)	0.18 (0.15-0.22)	0.14 (0.11-0.15)
T_{ancDOG}	0.15 (0.14-0.17)	0.23 (0.2-0.27)	0.25 (0.22-0.29)	0.24 (0.22-0.27)
T_{ancIB}	0.06 (0.03-0.08)	-	0.07 (0.06-0.08)	0.14 (0.13-0.16)
$T_{ancIT-IB}$	0.09 (0.06-0.11)	0.09 (0.04-0.13)	0.09 (0.07-0.11)	0.15 (0.13-0.16)
$T_{ancCR-IT-IB}$	0.1 (0.07-0.11)	0.1 (0.04-0.14)	0.1 (0.07-0.12)	0.15 (0.14-0.16)
$T_{ancIS-CR-IT-IB}$	0.3 (0.27-0.32)	0.24 (0.21-0.29)	0.2 (0.16-0.22)	0.2 (0.16-0.24)
$T_{ancWOLF}$	0.3 (0.28-0.32)	0.25 (0.22-0.31)	0.2 (0.17-0.22)	0.22 (0.17-0.26)
T_{ancDW}	0.32 (0.3-0.35)	0.31 (0.26-0.34)	0.31 (0.28-0.33)	0.3 (0.27-0.33)
T_{root}	13.99 (11.02-15.58)	14.61 (12.16-15.79)	12.88 (11-15.44)	13.34 (10.9-15.55)
$m_{BOX->SPW}$	29.33 (4.77-73.08)	-	0.01 (0-0.08)	12.82 (2.6-19.62)
$m_{BAS->SPW}$	6.29 (0-12.13)	-	0.02 (0-0.11)	1.66 (0-6.62)
$m_{DIN->SPW}$	2.85 (0-5.9)	-	0.02 (0-0.14)	0.55 (0-2.75)
$m_{BAS->Isw}$	7.03 (5.39-8.59)	5.72 (4.06-7.45)	6.01 (4.46-7.56)	8.11 (6.01-10.36)
$m_{ISW->DIN}$	4.15 (0-8.36)	5.39 (1.79-9.02)	3.82 (0-6.11)	4.43 (0-9.11)
$m_{PTW->ITW}$	4.6 (0-15.58)	3.32 (0-20.27)	2.49 (0-9.49)	0.73 (0-4.51)
$m_{ancDW->JAC}$	1.39 (1.3-1.46)	1.39 (1.32-1.46)	1.35 (1.28-1.42)	1.36 (1.26-1.44)
$m_{CHW->ancWOLF}$	19.28 (0-127.61)	64.63 (0-258.09)	30.54 (0-174.44)	32.41 (0-104.46)
$m_{ISW->CHW}$	0.28 (0-1.98)	0.13 (0-0.86)	0.77 (0-3.87)	0.55 (0-3.79)
$m_{DIN->CHW}$	2.88 (1.87-3.99)	2.07 (1.23-2.92)	2.16 (1.24-2.96)	2.01 (1.21-2.87)
